# A longitudinal, randomized experimental pilot study to investigate the effects of airborne infrasound on human mental health, cognition, and brain structure

**DOI:** 10.1038/s41598-021-82203-6

**Published:** 2021-02-04

**Authors:** L. Ascone, C. Kling, J. Wieczorek, C. Koch, S. Kühn

**Affiliations:** 1 Neuronal Plasticity Working Group, Department of Psychiatry and Psychotherapy, University Medical Center Hamburg-Eppendorf, Martinistraße 52, 20246 Hamburg, Germany; 2 Lise Meitner Group for Environmental Neuroscience, Max Planck Institute for Human Development, Lentzeallee 94, 14195 Berlin, Germany; 3 Physikalisch-Technische Bundesanstalt Braunschweig, Bundesallee 100, 38116 Braunschweig, Germany

**Keywords:** Neuroscience, Risk factors, Psychology

## Abstract

Airborne infrasound (IS; emitted by e.g., large machinery, wind farms) is ubiquitous in technologized environments. Health hazards are controversially discussed at present. This study investigated long-term effects of IS on brain (regional grey matter volume; rGMV) and behavior in humans. Specifically engineered infrasonic (6 Hz, 80–90 dB) vs. sham devices were installed in participants’ (N = 38) bedrooms and active for 28 nights. Somatic and psychiatric symptoms, sound-sensitivity, sleep quality, cognitive performance, and structural MRI were assessed pre-post. Null findings emerged for all behavioral variables. Exploratory analyses revealed a trend (*p* = .083) with individuals exposed to IS reporting more physical weakness at post-test (*d* = 0.38). Voxel-based morphometry (VBM) revealed no rGMV increases, but there were decreases within clusters in the cerebellum VIIIa (bilateral) and left angular gyrus (BA39) in verum. In conclusion, IS does not affect healthy individuals on a global scale. However, future trials should consider more fine-grained specific effects, combining self-report with physiological assessments, particularly directed at bodily sensations and perception. As no brain-behavior-links could be established, the identified grey matter decline cannot be interpreted in terms of potential harmfulness vs. improvement through IS-exposure. Parameters that may best reflect brain changes as established in the present study include motor function, sensory processing/ bodily- and motor-perceptions, working memory, and higher auditory processing (i.e., language-related tasks), which are hence potential target variables for further research.

## Introduction

In the wake of industrial and technological innovations of the twentieth century, an emerging question is whether sounds below the human hearing threshold (≈ 20 Hz), termed infrasound (IS), can affect human behavior and brain structure. It shall be noted that the hearing threshold, provided high sound pressure levels (SPLs), is variable, and a hearing sensation can be provoked at infrasonic frequencies. For instance, it has been demonstrated that 2.5 Hz can be perceived at a mean level of 120.7 dB SPL via a monaurally inserted earphone source^1^. Concerning adverse effects, symptoms like nausea, malaise, fatigue, aversion to the area, non-specific pain, sleep disturbances, or annoyance have been documented for IS, mainly in earlier, uncontrolled studies with industrial workers or observational field studies on individuals exposed to IS due to living in the vicinity of IS-sources (e.g., see^2–4^). Points of criticism concerning these studies are manifold. For instance, audible low-frequency (LF) components were usually involved, precluding a definite answer to whether adverse effects were attributable to inaudible IS or merely an effect of audible noise. More recent reviews take an accordingly skeptical tenor concerning adverse health effects directly induced by IS. One of these reviews suggested that psychological and social mechanisms may contribute to annoyance, which explains the observed adverse health effects better than exposure to infra- or low-frequency noise per se^5^. According to another review, annoyance is reported by about 10% of individuals living in proximity to relevant infrasonic or low-frequency sources^6^. Both reviews correspond in their call for methodologically well-designed long-term studies, which are lacking at present.

Concerning potential brain effects of IS, not to mention inaudible IS, the literature is extremely scarce. In a first ground-breaking study by Dommes and colleagues^7^, exposure to low frequency and IS was associated with an altered BOLD response in the primary auditory cortex and superior temporal gyrus (an area largely responsible for higher-order auditory processing, such as language comprehension). The signal was evident at SPLs between 90 and 110 dB for audible infrasonic and low frequencies between 12 and 500 Hz^7^. Another pilot study conducted by our research group found that IS generates brain activities within the auditory cortex down to a frequency of 8 Hz^8^, if IS is presented at SPLs above the hearing threshold. A further study investigated resting-state brain activity and found it to be significantly altered by near-threshold (hence inaudible) IS at a frequency of 12 Hz. Higher local connectivity in the right superior temporal gyrus (primary auditory cortex), the anterior cingulate cortex, and the right amygdala, was identified. This speaks for the possibility of inaudible IS provoking attentional alterations, physiological changes, and stress responses^9^. Somewhat contradicting the idea of IS inducing adverse effects, in the same project, our research team also found a trend-level improvement in working memory performance under the influence of perceivable 12 Hz IS^10^. Also slightly unexpected were the results of a study investigating the interaction of IS with audible sound. While IS does not alter hearing thresholds at audible frequencies, vice versa audible noise increased the thresholds of IS tending to reduce the load of IS on humans in an exposure situation^11^.

Despite some prima-facie evidence of potential effects of IS on human (mental) health and cognition, there are virtually no studies that directly investigated the effect of inaudible IS on human behavior in a randomized-controlled fashion, and there is so far no study of its effects on brain structure. There is plenty of evidence by now, showing that our immediate environment and lifestyle can have a profound impact on the brains’ morphology, and IS sound is now ubiquitous in our environment. It emerges from different sources, just to name a few: plain traffic, large ventilation systems, public transportation, wind farms, heat pumps, or large machinery^12^. So far, all designed laboratory studies have used short signals as stimuli which do not match the real situation of exposure to an IS source in the public and all-day life. The present study is the first ever-conducted randomized long-term exposure trial (one month) of humans to airborne inaudible IS vs. placebo, addressing this issue in an exploratory and exhaustive manner. Effects of inaudible IS on human mental health (i.e., psychiatric symptoms in general, anxiety, depression, stress), somatic symptoms (i.e., sleep disturbances, somatic symptoms), cognition/ attention (i.e., alertness, vigilance, cognitive flexibility, divided attention, attention shifting, inhibition), and brain structure have never been studied comprehensively before, which is the intention of the present study.

## Methods

### Recruitment and in- and exclusion criteria

The trial was pre-registered in the National Institute of Health trial registry (full trial protocol available here: https://clinicaltrials.gov/ct2/show/NCT0345918; trial identifier: NCT03459183, ID: SonicBrain01; registration date: 08/03/2018). In all procedures, we adhered to the declaration of Helsinki, and the study was approved by a local ethics board prior to study onset (Ethik-Kommission der Ärztekammer Hamburg; approval number: PV5570). Active recruitment and data collection took place between May 31, 2018 and December 15, 2019. We obtained informed consent from all study participants who were enrolled in the study. The study comprised a 2 (infrasound verum vs. placebo) × 2 (pre-post one month of sound exposure) repeated-measures randomized-controlled, single-blind (participants were unaware of group assignment) design. Participants were assigned to the conditions based on list-wise randomization, with an allocation ratio of 50:50. The randomization list included a computer-generated random sequence which was implemented by the first author (L.A.), and restrained to 25 slots per condition (taking into account potential drop out). Included participants were sequentially assigned to the next available list position and unaware of their assignment until the end of the trial. The experimenter was aware of the group assignment. Several advertisements in local newspapers were run, and flyers systematically spread across the city of Hamburg, searching for healthy test persons. Interested individuals who contacted the study team first received exhaustive study information and a link for an online screening to check in- and exclusion criteria. The screening took about 45 min, and included socio-demographic assessments, including sex, age (required to be between 18 and 45 years), education, partnership status, children, regular medication intake, and variables addressing housing conditions (incl. size of the bedroom, number of windows and doors in the bedroom, city district, and closeness to main roads). Children sleeping in the bedroom was an exclusion criterion for safety reasons. In addition, pet owners were advised to keep animals outside the room for the time of the exposure. Main exclusion criteria were counter-indications for magnetic resonance imaging (MRI) (i.e., cochlear implants, non-removable metal on/ in the body, or tinnitus), chronic inflammatory, autoimmune, or other severe illnesses (e.g., cancer), as well as central-nervous-system diseases. Similarly, indicating any anomalies concerning hearing (e.g., deafness, past ear surgery, chronic inflammation of the ear canal, chronic sinusitis, anatomic anomalies) lead to exclusion. Central nervous medication intake or participation in a concurrent medical trial also led to exclusion. Further health-relevant variables assessed were smoking and alcohol consumption. Mental illness was assessed using standardized screening tools: the Mini International Neuropsychiatry Interview (MINI)^13^ for axis I disorders, and the Structured Clinical Interview for DSM-IV – II (SCID-II)^14^ for axis II (personality) disorders. Only the screening questions of the respective interviews were included in the online survey. Any indication of a mental disorder was followed up on in a subsequent telephone interview, which, depending upon the amount of positively endorsed clinical screening questions, could take between 20 and 60 min. Telephone contacts also included providing further information on the study and answering the participants’ questions. Medical or psychological student research assistants, who were trained and supervised by a postdoc level clinical psychologist, conducted all telephone screenings. Suspicion of a potential mental disorder led to exclusion from the study.

A minimum total sample size of N ≈ 40 was determined based on previous experience of the principal investigator (S.K.), who is an expert in conducting research of neuroplasticity induced by environmental changes that were observed in comparable samples after experimental interventions between 4 and 8 weeks. This constituted a minimum compromise based on available time and resources.

### Study procedure

If all in- and exclusion criteria were fulfilled, appointments for the pre-test, on-site sound source installation, and post-test were made. Both assessments before and after the exposure took place at our research laboratory unit at University Medical Center Hamburg-Eppendorf. Each assessment was divided into two blocks: the first block included questionnaire assessments (self-reports of somatic and mental illness symptoms, sleep quality self-reports, personality tests) intermixed with computerized cognition tests (e.g., alertness, inhibition, task switching, working memory, sustained attention), (total duration between 2 and 2.5 h). The second block included an MRI session (total duration between 1 and 1.5 h). As part of the MRI session, individuals also performed a spatial n-back task.

Closely after the pre-test assessment (1 day to maximally one week after initial assessment), the on-site sound source installation took place. For the entire process, we followed a standardization of procedure protocol (see Appendix I for details). The IS sources were configured in such a way that they would emit a steady SPL between 80 and 90 dB (6 Hz frequency) for eight hours during the participant’s self-reported habitual sleep (bed) time interval (see Appendix I for details). We chose this range of SPL for two reasons. For one, it can be expected to be below the hearing threshold, necessary for a blind study but second, 80 dB to 90 dB SPL can already be considered as a high acoustic load, hence enhancing the likelihood of finding (if present) any effect. The SPL range chosen is about 25 dB higher than common IS emissions from wind parks (see for example^15,16^). Other potential sources may generate a wide range of IS noise levels, but 80 dB to 90 dB SPL seemed well adapted to common exposure situations^17,18^. The choice of 6 Hz was a compromise between technical issues of source manufacturing (the lower the frequency the bulkier is the design) and the wish to have a signal with low frequency. The sham sources looked and operated identically to the verum sources but did not emit any sound. For a detailed description of the design, technical details, and initial calibration of the IS sources, and for descriptive data on the on-site constellation and exposure levels please refer to Appendix I. After the exposure, the post-test, with the same measures, taken in the same order as at pre-test, took place.

### Measures

#### Self-reports

All self-reports were assessed always in the same order for all participants at all assessment points (baseline [pre-test], post-test) and filled out by the participants on a computer.

Normal (hearing sound) sensitivity was assessed using the Noise Sensitivity Questionnaire which has been reported to have excellent reliability (0.90)^19^. The questionnaire comprises 35 items rated on a 4-point Likert scale (*strongly agree* = *3, slightly agree* = *2, slightly disagree* = *1, and strongly disagree* = *0*). Sensitivity to high-frequency sound in particular was assessed using the SISUS-Q (sensitivity to infra- and ultrasound questionnaire) which is a brief scale consisting of four items rated on an 11-point Likert (*0* = *totally disagree, 10* = *totally agree*), which assesses high-frequency-sensitivity with good reliability (Cronbach’s alpha = 0.85)^20^. Both normal hearing sound and high-frequency sensitivity were assessed at pre- and post-test and groups were checked for differences in these variables at baseline in order to preclude bias of results by differences in sensitivity.

The Brief Symptom Inventory (BSI)^21^ was used to measure global severity of psychiatric symptoms. It contains 53 items, asking for how strongly respondents were affected (*not at all* = *0*; *extremely* = *4*) by a range of different problems, which can be categorized into nine symptom group subscales. We separately analyzed the somatization, depression, and anxiety subscales. The BSI has been shown to have sufficient to excellent reliability with Cronbach’s α of 0.90 for the global severity index, 0.63 for somatic symptoms, 0.62 for anxiety, and 0.72 for depression. Participants were instructed to rate symptoms for the past 2 weeks.

The Perceived Stress Scale (PSS) measures the perceived stressfulness of daily life situations. Fourteen items address how often the respondents felt stressed (vs. in control of things) on a 5-point frequency scale ranging from 0 (= *never*) to 4 (= *very often*). Reliabilities (Cronbach’s α) have been reported as good (0.84–0.86)^22^. Again, participants answered the questions referring to the last two weeks.

In order to assess daytime sleepiness and fatigue, the Epworth Sleepiness Scale (ESS) was used^23^. It asks the participant to rate the perceived likelihood of dozing in eight typical daytime activities (e.g., sitting quietly after a lunch without alcohol; *would never doze* = *0*, *slight chance of dozing* = *1*, *moderate chance of dozing* = *2*, *high chance of dozing* = *3*). The scale refers to daily life in recent time. Good reliability (Cronbach’s α of 0.88) has been reported^24^.

Overall, quality or disturbances of sleep was assessed using the Pittsburgh Sleep Quality Inventory (PSQI)^25^. Seven components, based on the participants’ replies to 19 questions, are evaluated: subjective sleep quality, sleep latency, sleep duration, habitual sleep efficiency, sleep disturbances, use of sleeping medication, and daytime dysfunction. The scores can range between 0 and 21, as each component is rated from 0–3, with lower ratings indicating poorer sleep quality. The components are usually integrated into a single global sleep quality score. The PSQI has been shown to sensitively differentiate between good and poor sleepers^25^. Reports in our study referred to the last two weeks.

Particularly neuroticism and introversion have been shown to be related to higher sensitivity, perceived loudness, and annoyance induced by high or low-frequency noise (e.g.,^26^). Hence, in order to make sure that there were no differences in this variable between the groups at baseline, a 30-item version of the five-factor personality inventory (NEO-FFI-3);^27^ was used. This questionnaire assesses *neuroticism* (characterized by ‘moodiness’ and frequent experience of aversive emotions), *extraversion* (enjoying human interactions, enthusiasm and zest, talkativeness, assertiveness, and gregariousness), *conscientiousness* (orderliness, self-discipline, dutifulness, competence, achievement striving, and deliberation), *openness* (intellectual curiosity, aesthetic sensitivity, attentiveness to feelings, preference for variety) and *agreeableness* (warmth, kindness and empathy). All items are rated on a 5-point Likert-scale, ranging from 0 (= *strongly disagree*) to 4 (= *strongly agree*). Both factorial validity and good internal consistencies (Cronbach’s α) have been reported for all subscales, ranging between 0.78 and 0.86^27^.

#### Cognition

We used the computer-based Tests of Attentional Performance (TAP)^28^ to assess a set of cognitive performance indicators in several domains, namely alertness, sustained attention, flexibility, divided attention, incompatibility (Simon task), covert shift of attention, and inhibition (GoNogo). For each test, different parameters are of relevance (see Appendix II).

#### MRI scanning parameters

Brain scans were performed with a 3 T Siemens Magnetom Prisma (Siemens Medical Systems, Erlangen, Germany) using a 64-channel head coil. A sagittally oriented 3D MPRAGE was run with 256 slices per slab, FOV = 240 mm, TR = 2500 ms, TE = 2.12 ms, TI = 1100 ms, voxel size = 0.8 mm × 0.8 mm × 0.9 mm.

### Statistical analyses

#### Voxel-based morphometry

We performed our pre-processing and whole-brain analyses using the toolboxes SPM12 (https://www.fil.ion.ucl.ac.uk/spm/software/spm12), (v7487) and CAT12 (Structural Brain Mapping Group, University of Jena; exact version: CAT12.6-rc1 [r1429] from 2019-02-08) (http://www.neuro.uni-jena.de/cat/index.html). We run the toolboxes with Matlab R2017a (MathWorks Inc., Natick, MA). Pre-processing steps were conducted following the default CAT12 segmentation routine for longitudinal data (http://dbm.neur.uni-jena.de/cat12/CAT12-Manual.pdf), which includes registering the segmented images to the MNI space using the high-dimensional Dartel approach.

#### Behavioral data analysis

A series of classical test theory repeated-measures ANOVAs were carried out in SPSS 25 (IBM Corp. 2017) for all 21 variables of interest [i.e., low-frequency sensitivity, normal sound sensitivity, psychiatric symptoms (total), somatization, depressive symptoms, anxiety, daytime sleepiness, sleep quality, perceived stress, alertness (4 indicators), sustained attention (1 indicator), flexibility (2 indicators), divided attention (1 indicator), incompatibility (2 indicators), covert shift of attention (1 indicator), GoNoGo (inhibition, 1 indicator variable)]. Post-hoc exploratory paired t-tests were applied to identify within-group changes underlying the interaction. To adjust for multiple testing, we used Bonferroni correction. Effect size *η*^2^_*partial*_ was interpreted as *η*^2^_*partial*_ > 0.01 small, > 0.06 medium, > 0.14 large effect.

#### Structural brain data analysis

We performed a whole-brain voxel-based morphometric (VBM) analysis with no prior assumptions concerning affected regions of interest (ROIs), as no pre-assumptions could be made due to the absence of research on structural brain effects of long-term IS exposure. The analyses were run with the preprocessed, segmented grey matter images using SPM12, examining both global increases and decreases in regional grey matter volumes (rGMV) in the IS verum condition, while controlling for, and assuming stability (no change) in the IS placebo condition (contrast increase in verum relative to placebo: −1 3 −1 −1; contrast decrease in verum relative to placebo: 1 −3 1 1). The same set of contrasts was computed for regional white matter volume (rWMV). As this was not the initial focus of the study, according findings can be found in detail in Appendix IV. A flexible factorial design was chosen, establishing a model with group and time factors and their interaction. An absolute threshold masking with a value of 0.01 was set. The resulting maps were thresholded with *p* < 0.001. The statistical cluster extent threshold was applied to correct for multiple comparisons. The latter was combined with a non-isotropic smoothness correction based on permutation as proposed by Hayasaka and Nichols^29^, (as implemented in the CAT12 toolbox).

#### Association of structural with behavioral changes

In a last step, we extracted mean GMV data from any identified significant clusters from the VBM analyses using the REX toolbox (release alpha 0.5; Neuro Imaging Tools and Resources Collaboratory; https://www.nitrc.org/projects/rex). The identified significant clusters (spmT-extent thresholded-cluster images) from the VBM were used as masks to extract the volumetric information within each of the clusters, separately for baseline and post-exposure assessments. Afterwards, we correlated the changes in volumetric rGMV data for each identified cluster with changes in behavioral data of variables that exhibited a significant change within verum. For variables differing from normality (skew and/ or kurtosis > 2 or < -2), and variables measured at a ranked, rather than interval level (e.g., number of errors), non-parametric correlations were computed (Spearman), as these have additionally been shown to be more robust in case of outliers^30^. For the correlations, we used Cohen’s^31^ rule of thumb to determine effect size: *r* ≥ 0.10 = small effect, r ≥ 0.30 small effect and *r* ≥ 0.50 = large effect.

### Ethics and participant consent

The study was approved by a local ethics consortium prior to study onset. The study adhered to the declaration of Helsinki. All participants consented to participate in the study.

## Results

### Sample

In total, 38 participants fully took part in the study, hence 38 pre-post datasets were available for analysis (*n*_verum_ = 23, *n*_placebo_ = 15). On top of these participants, there were 5 dropouts (4 during pretest and before group allocation, one after group assignment and sound source installation). Reasons for dropouts at pre-test were claustrophobia in the scanner (3 cases), and mental disorder (1 case) that was revealed during pre-test. The dropout after baseline (completed pre-test) was due to pregnancy (1 case—exclusion after sound source installation—placebo group). Socio-demographic details for each group are shown in Table 1. There were no significant differences in any of the demographic variables across the groups at baseline. There were no adverse events leading to premature study termination. There were no baseline differences between the groups in any of the personality dimensions (neuroticism, openness, conscientiousness, extraversion, agreeableness) or sensitivity (normal sound, low frequency; all *p* > 0.10; for descriptive data of these variables see Appendix III).

**Table 1:** Descriptive sample data and between-group differences for socio-demographic variables.

Variable/descriptives	Infrasound—verum (*n* = 23)	Infrasound—placebo (*n* = 15)	Inferential statistics
Age: mean (SD)	27.35 (6.44)	25.60 (4.76)	*t*(36) = 0.90, *p* = .373
Sex: percentage male/female (no. male/no. female)	43.5/ 56.5 (10/13)	33.3/ 66.7 (5/10)	*X*^*2*^(1, 38) = 0.39, *p* = .532
Years of education	15.26 (3.97)	15.40 (1.81)	*t*(36) = 0.13, *p* = .900
Children: percentage yes/no (no. yes/no)	17.4/ 82.6 (4/19)	6.7/ 93.3 (1/14)	*X*^*2*^(1, 38) = 0.91, *p* = .339
Nationality: percentage German/other (no. German/ other)	91.3/ 8.7 (21/2)	93.3/ 6.7 (14/ 1)	*X*^*2*^(1, 38) = 0.05, *p* = .821
Regular medication: percentage yes/ no (no. yes/no)	13.0/87.0 (3/20)	20.0/80.0 (3/12)	*X*^*2*^(1, 38) = 0.33, *p* = .565

### Behavioral results

For descriptive pre-post data please refer to Appendix III. All repeated measures ANOVA main results can be found in Table 2. All analyses were carried out with the original groups as assigned. Given that *N* = 21 hypothesis tests were carried out, the rate of positive results (H_1_) identified based on chance (false positive rate) is 21 × 0.05 = 1.05. The identification of more than one significant result may indicate that one of these findings is genuine. One significant interaction with a medium effect size (*η*^2^_*partial*_ = 0.120, *p* = 0.033) identified for the variable *somatization*. Follow-up paired t-tests revealed that this effect was attributable to a significant, medium-sized decrease of somatic symptoms in the placebo condition *t*(22) = − 2.25, *p* = 0.027; *d* = 0.64 (no significant change in verum; *t*(14) = 0.93, *p* = 0.365; *d* = 0.19). Bonferroni-correction sets the significance needed to reject the H_0_ at *p* < 0.0024, hence the identified effect does not survive multiple comparison correction. As the somatization scale of the BSI assesses heterogeneous symptoms, exploratory single-item-based analyses were carried out, checking for somatic within-group changes. Vertigo, chest pain, nausea, respiratory problems, heat/chills, numbness/tingling in parts of the body, did not change significantly (*p* > 0.10) within any of the groups individually. However, weakness perceived in parts of the body increased at trend-level within IS verum (*t*(22) = 1.82, *p* = 0.083; *d* = 0.38) and dropped significantly within IS placebo (*t*(14) = 2.65, *p* = 0.019; *d* = 0.68).

**Table 2:** Results (group x time interaction effects) of the repeated measures ANOVAs for all behavioral variables.

Dependent variables	Statistics for the interaction effect
**Sensitivity**	
Low frequency sensitivity	*F*(1,36) = 0.59, *p* = .449, *η*^2^_p_ = .016
Normal sound sensitivity	*F*(1,36) = 0.03, *p* = .867, *η*^2^_p_ = .001
**Symptoms and sleep**	
BSI total	*F*(1,36) = 0.05, *p* = .823, *η*^2^_p_ = .001
BSI somatization	***F(1,36) = 4.89, p = .033, η***^**2**^_**p**_** = .120**
BSI depressive symptoms	*F*(1,36) = 1.12, *p* = .297, *η*^2^_p_ = .030
BSI anxiety symptoms	*F*(1,36) = 0.80, *p* = .377, *η*^2^_p_ = .022
ESS sleepiness	*F*(1,36) = 4.08, *p* = .051, *η*^2^_p_ = .102
PSQI—sleep quality (total)	*F*(1,27) = 1.37, *p* = .252, *η*^2^_p_ = .048
PSS perceived stress	*F*(1,36) = 2.28, *p* = .140, *η*^2^_p_ = .060
**Alertness**	
Median RTs tonic arousal	*F*(1,36) = 1.06, *p* = .309, *η*^2^_p_ = .029
Median RTs phasic arousal	*F*(1,36) = 0.02, *p* = .887, *η*^2^_p_ = .001
Phasic alertness index	*F*(1,36) = 0.62, *p* = .437, *η*^2^_p_ = .017
Anticipations in tone condition	*F*(1,36) = 3.51, *p* = .069, *η*^2^_p_ = .089
**Sustained attention (WM)**	
Omissions (total)	*F*(1,36) = 0.59, *p* = .448, *η*^2^_p_ = .016
**Flexibility**	
Speed-accuracy index	*F*(1,36) = 2.42, *p* = .129, *η*^2^_p_ = .063
Total performance index	*F*(1,36) = 0.38, *p* = .540, *η*^2^_p_ = .011
**Divided attention**	
Omissions (total)	*F*(1,36) = 0.04, *p* = .841, *η*^2^_p_ = .001
**Incompatibility**	
Incompatibility effect (visual field × hand)	*F*(1,35) = 2.22, *p* = .145, *η*^2^_p_ = .060
Errors incompatible	*F*(1,36) = 1.03, *p* = .316, *η*^2^_p_ = .028
**Covert shift of attention**	
Validity × side (re-orientation of attention)	*F*(1,35) = 2.53, *p* = .121, *η*^2^_p_ = .067
**GoNoGo (inhibition)**	
Errors (total)	*F*(1,36) = 0.19, *p* = .669, *η*^2^_p_ = .005

Alpha level = 0.05; Bonferroni-adjusted = 0.05/21 = 0.024. After application of the corrected *p*-level, the significant effect (with unadjusted level, highlighted in bold in Table 2) would not remain. The likelihood of by chance detecting a significant result is 5%, with 21 tests this equals 1.05 tests that would be detected as significant by pure chance.

### Brain structure results

**Figure 1:**
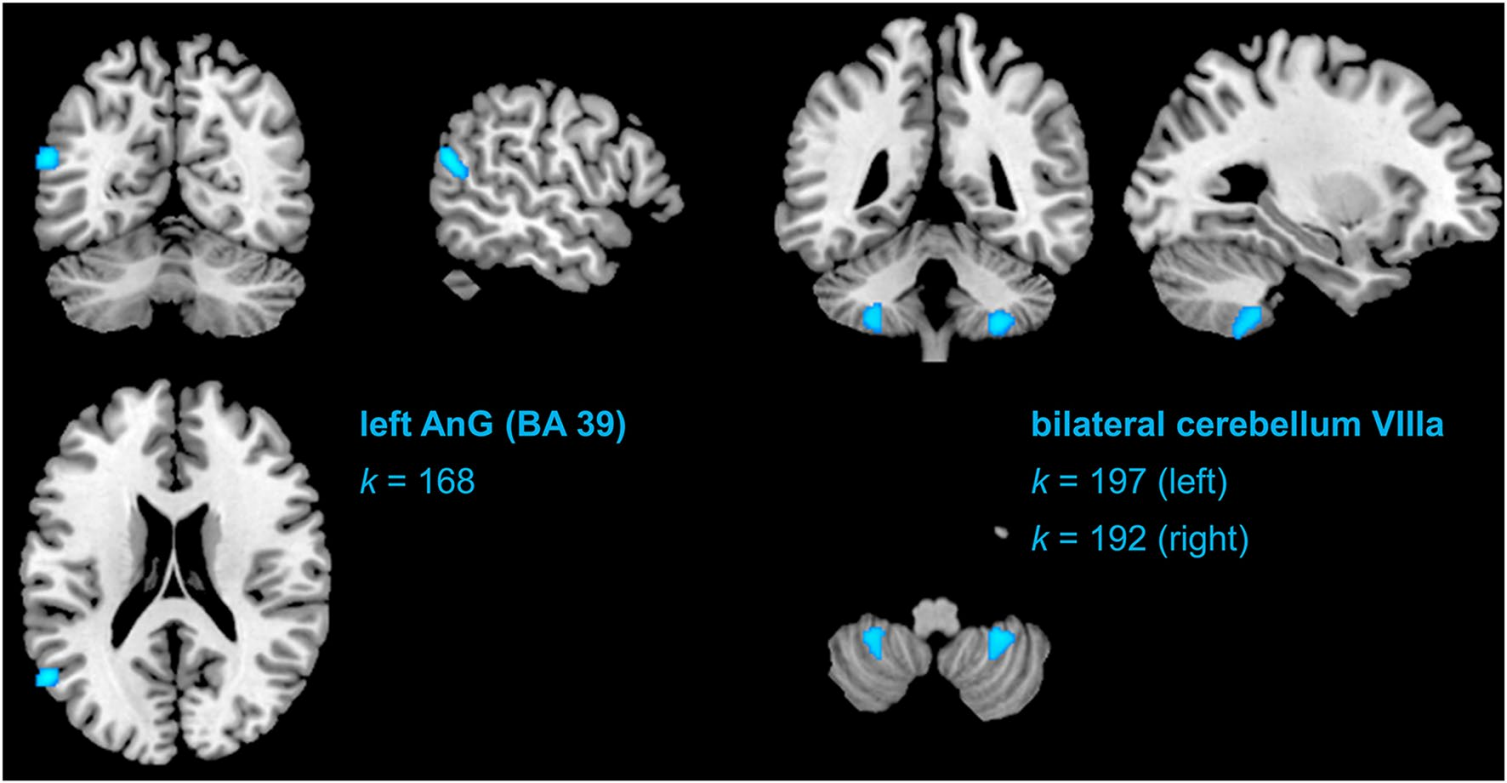
Graphical depiction of identified significant clusters in the VBM analysis searching for decreases in rGMV in the infrasound verum condition (relative to the sham [placebo] condition) from pre-to-post.

Complete structural pre- and post-test data was available for all participants in verum (*n* = 23) and placebo (*n* = 15). There were no clusters identified hypothesizing an increase in verum relative to placebo (cluster size extent threshold *k* > 96). We identified three clusters of significant pre-to-post decrease in the IS verum condition (contrasted with placebo; cluster size extent threshold *k* > 96): (1) a cluster mainly corresponding to the superior temporal gyrus (STG; BA39 [angular gyrus]; − 56, − 62, 20; *t* = 4.74, *k* = 168), and (2), (3) bilateral clusters in the exterior cerebellum (left cerebellum VIIIa: − 29, − 44, − 51, *t* = 4.19, *k* = 197; right cerebellum VIIIa: 29, − 45, − 53, *t* = 4.35, *k* = 192). The identified clusters are depicted (at their peak intensity coordinate) in Fig. 1. Overall, the findings indicate that only a decrease model applies to the structural rGMV data for IS.

### Correlations between behavioral and regional grey matter volume changes

Change scores of the identified clusters of rGMV decrease were correlated with one another within the IS verum group, with the following results: there was a significant association between the decreases in rGMV in right cerebellum (VIIIa) and left angular gyrus (STG), *r*(23) = 0.592, *p* = 0.003. The bilateral decreases in cerebellum (VIIIa) were not significantly related to one another, *r*(23) = 0.203, *p* = 0.420, nor were decreases in left cerebellum related to the decreases observed in left angular gyrus, *r*(23) = − 0.217, *p* = 0.319. Changes in somatization within in the IS verum group were correlated with rGMV changes: there were no significant change-to-change correlations (changes in rGMV—angular gyrus with somatization: *rho* = 0.128, *p* = 0.562;—right cerebellum VIIIa with somatization: *rho* = − 0.149, *p* = 0.496;—left cerebellum VIIIa with somatization: *rho* = − 0.289, *p* = 0.182).

## Discussion

To our knowledge, the present study was the first-ever conducted randomized-controlled longitudinal exposure trial, designed to answer the question of whether inaudible sounds below the human hearing threshold (*f* < 20 Hz), commonly known as infrasound (IS), affect human (mental) health, cognition and brain structure. Our results have important implications concerning target variables that are affected by IS. Discussion of key findings follows in the next paragraphs.

### Effects of infrasound on human behavior

Our results suggest that inaudible IS has no impact on a broad range of behavioral variables in healthy, young volunteers, including self-reported health (somatic, psychiatric), sleep, and computer-assessed cognition parameters (including alertness, flexibility, sustained attention [vigilance], inhibition, shifted attention). Exploratory analyses suggest that IS may evoke feelings of weakness to a small extent, but the evidence is based on single-item exploratory post-hoc analysis and warrants thorough replication.

### Effects of infrasound on brain structure and correlations with behavior

In the whole-brain voxel-based morphometry analyses, there were bilateral reductions of rGMV within clusters of the cerebellar VIIIa region in the IS verum condition (relative to placebo) from pre-to-post. Usually, this area and other parts of the cerebellum are involved in motor function, but recent evidence also suggests an important role in cognition, such as visual working memory (^32^). Moreover, research has evidenced a general paradigm shift concerning the role of the cerebellum as involved in cognitive function^33^, which is of importance in interpreting the present findings. The majority of the cerebellum contralaterally maps to the association cortex in a topographically ordered manner, whereby it has been hypothesized that the cerebellum contains two mirrored representations of the cerebral cortex^33^. When it comes to the identified clusters in our study, the mapped areas within the cortex would roughly correspond to the somatomotor cortex, hearing- and language-related areas (including angular gyrus and primary somatosensory cortex of the eardrum), as well as areas of executive functioning (incl. working memory). The finding of a significant cluster of rGMV decrease in the left superior temporal gyrus (angular gyrus, BA39), also being related to the significant decline in the right cerebellum VIIIa, speaks for a potential decline in higher auditory processing. Speech-related functions, such as lexical, semantic, and phonological (discrimination) tasks, reading or speech production, and intelligibility are important behavioral candidate target variables for future study. Corroborating this claim, speech intelligibility has been found to be impaired through IS in earlier studies (e.g.,^34^). At least on a level of face validity, the rGMV reductions in angular gyrus also align with findings of Persson-Waye and colleagues^35^, who found worse performances in a grammatical performance and proofreading task in an infrasonic noise relative to a control group, although the authors used a different, and partly audible, sound stimulus. In addition, our results fit with the first fMRI study on low-frequency noise and IS by Dommes and colleagues^7^, which demonstrated altered BOLD responses in the superior temporal gyrus. The study, however, could not clearly exclude that higher harmonics generated by the non-perfect source were above the hearing threshold within the audible frequency range.

Finally, in the present study, there were no significant links between behavioral (somatization) and structural brain changes, although there was a trend-level exploratory finding of individuals perceiving more unspecific bodily weakness and cerebellar decline would possibly suggest sensory- or somatomotor changes. Hence, more fine-grained assessments of bodily sensations, combined with physiological parameters (e.g., electromyography) and, potentially, other perceptual changes (e.g., eardrum pressure) would be recommendable for future studies.

### Limitations

We did not select our participants according to self-reported sensitivity for IS, which may have led to an underestimation of potential effects. In addition, our sample was rather of young age. Follow-up investigations will need to recruit a broader, more balanced age-range and include individuals with self-reported high-sensitivity. In addition, expectation effects need to be addressed more thoroughly, as there is evidence for nocebo responses after receiving corresponding information about negative effects of extreme frequencies such as wind turbine noise^36^.

## Conclusion

Our study broadly suggests that inaudible (6 Hz) IS does not affect human behavior per se, including a range of health-related and psychological variables (i.e., self-reports of sound sensitivity, sleep, psychiatric symptoms, or stress) and cognitive functions (i.e., alertness, sustained attention, cognitive flexibility, divided attention, shift of attention, inhibition). Based on our brain structural analyses, it seems that IS exposure relates to grey matter decline in brain areas that are associated with somatomotor- and cognitive functions such as working memory (bilateral cerebellum VIIIa) and higher auditory processing (angular gyrus, BA39), comprising functions such as speech intelligibility/production or semantic/lexical processing and reading. The overall pattern of results, including exploratory findings of changed body perception (increased perceived weakness), makes a plausible case for assessing bodily sensations at a level of greater detail, adding physiological assessments such as electromyography, and to focus on tasks related to language processing and complex (verbal) working memory in future trials.

## Supplementary Information

Supplementary Information.

## Data Availability

We hereby declare that our data, code and syntaxes (including a documentation of all analyses that were undertaken) are available upon request.
