# Supplementary material for: A longitudinal, randomized experimental pilot study to investigate the effects of airborne infrasound on human mental health, cognition, and brain structure
Source: Sci Rep. 2021 Feb 4;11:3190. doi: 10.1038/s41598-021-82203-6 (PMC7862356; doi:10.1038/s41598-021-82203-6)
Supplement: Supplementary file 1 — Supplementary Information. [file 41598_2021_82203_MOESM1_ESM.pdf]

A Longitudinal, Randomized Experimental Pilot Study to Investigate the Effects of Airborne Infrasound on Human Mental Health, Cognition, and Brain Structure

APPENDIX

Ascone, L.[1], Kling, C.[3], Wieczorek, J.[3], Koch, C.[3], Kühn, S.[1,2]

[1] University Medical Center Hamburg-Eppendorf, Department of Psychiatry and Psychotherapy, Neuronal Plasticity Working Group, Martinistraße 52, 20246, Hamburg, Germany.

[2] Max Planck Institute for Human Development, Lise Meitner Group for Environmental Neuroscience, Lentzeallee 94, 14195 Berlin, Germany

[3] Physikalisch-Technische Bundesanstalt Braunschweig, Bundesallee 100, 38116 Braunschweig, Germany

Contact information of corresponding author: l.ascone-michelis@uke.de

Appendix I

1a) Design, technical details and initial tests of infrasound sources

The infrasound sources were specifically developed for this study. Six sources were assembled in total, three fully functional sources for the verum group and three dummy sources for the placebo group. The sources were designed to be installed by the setup team in the participants bedroom and then to work for several weeks autonomously and reliably. The working principle was based on an acoustical resonance similar to a transmission line box. The main components were a frequency generator, producing a 6 Hz sinusoidal tone, an amplifier, a loudspeaker, and a 26 m flexible tube attached to the speaker (see Appendix Figure 1). The outer dimensions were 65 cm (length) x 65 cm (width) x 104 cm (height). The case was made of medium-density fiberboard (MDF). Appendix Figure 2 shows a photograph of one of the sources.

Essentially, the source is a driven active loudspeaker. The requirement for a very pure low-frequency high-level sound emission gave the need for a sophisticated design. By combining several electrical low-pass filters with the tube’s acoustical resonance, it was ensured that the source produced a proper infrasound signal without any audible harmonic components or hum. A large air volume of about 0.4 l to 0.8 l had to be moved rapidly inside the source to produce the desired sound pressure level at a frequency of 6 Hz which led to the situation that any edge in the sound canal produced strong wind noise. The design considered that issue by avoiding any irregularities inside and by adapted sound outlets. The heat produced by the electrical components inside the source was regulated by a specific temperature control and cooling system designed to operate with no emission of audible noise. The case was designed in a way to ensure undisturbed cooling and sound emission even if the source would be placed close to furniture or other neighboring structures. Elastic mounting minimized structure-borne sound transmission. The sources required only a standard plug socket for 230 V power supply and were simply switched on and off without any other functionality. For the bedroom

insonification, a standard time switch turned them on and off. Several LEDs on the front panel gave feedback on the operating status of the source. The placebo sources were designed to apparently behave and appear in the same way as the verum sources; thus, the participants were not able to distinguish between the two kinds of sources.

The sources were preliminarily tested in the laboratory in rooms with different volumes and dimensions typical for bedrooms. Sound pressure levels between about 80 dB and 100 dB (ref. 20 µPa) were produced dependent on the room's volume, its geometry, the opening state of the room's windows and doors and the acoustic coupling to the neighbor rooms. In general, the sound pressure is higher in small volumes, with cube-like dimensions and windows slightly opened. To handle these physical effects in the field tests, the setup team adjusted the sound pressure level in an individual bedroom by tuning the amplification in the source. A sound level meter Ono Sokki LA-215 was used for the sound pressure measurement at site, which was tested and calibrated before the start of the field study.

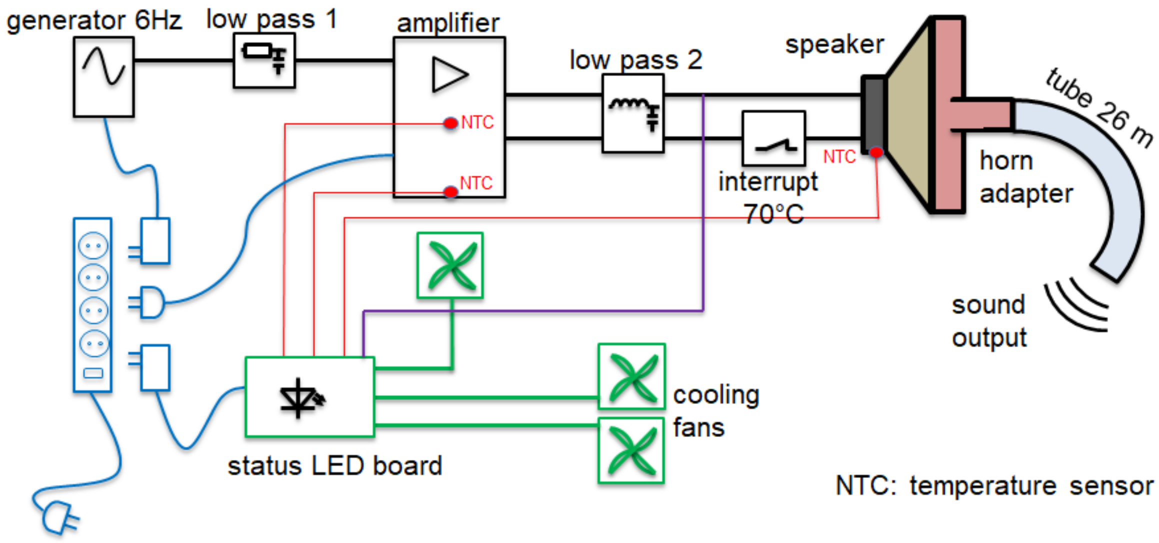

Appendix Figure 1. Block diagram of the infrasound source.

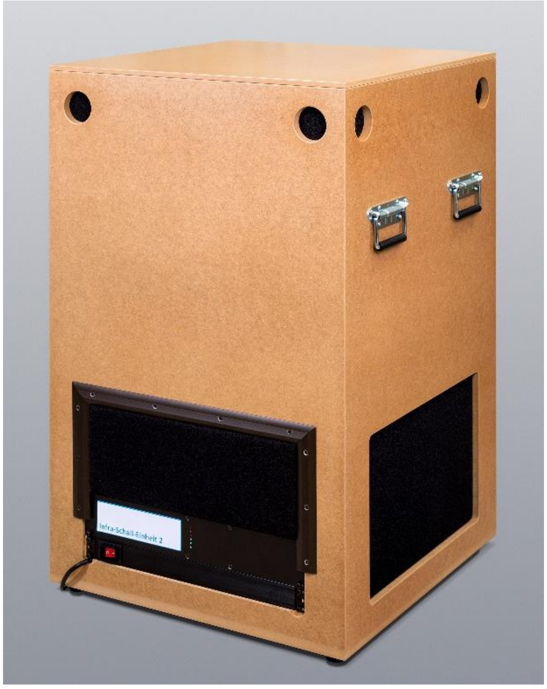

Appendix Figure 2. Photograph of an infrasound source

1b) Description of standardized on-site measurement and adjustment protocol

All measurements on-site in the infrasound verum group (in the participants' bedrooms) were taken after 6 pm (at 6:45 pm on average), with one exception due to a shift work situation (measurements taken at 10:30 am). Similarly, in the infrasound placebo group, all measures (background SPL) were taken at 6:23 pm on average, with one exception due to work shifts and the impossibility to find another appointment (measurements taken at 1:30 pm). Measurements were implemented with a sound level meter (Ono Sokki LA-215), with configurations to cover the entire frequency spectrum as measurable with this device and hence capturing the SPL of the background level vs. SPL with the switched-on infrasound verum sources. The SPL was measured as the sound pressure level with frequency weighting Z (unweighted or flat weighting) and time weighting FAST. In order to realize the measurements in a standardized manner, we developed a standardization of procedure protocol, which we briefly describe in chronological order in the following paragraph.

(1) First, it was explained to all participants that we would install and calibrate the sound source in their bedroom, which would require them to leave the room later in the process. Next,

they were interviewed concerning their typical sleep interval and about potential additional noise sources in the night. Their preferred and most frequent sleep constellation was assessed (i.e., position of doors and windows). We communicated to them that an 'all-closed' constellation would be preferable for reasons of controllability of the experiment, however, if participants communicated that they clearly preferred other constellations and would consistently use them for the duration of 28 days, this was tolerated, and measurements taken for this particular constellation. In addition, a second-favorite and frequent constellation was asked for. The participants were now sent out of the room, asking them to close the door, while explaining to them that absolute quiet was needed for the measurement procedure.

(2) We now measured the room (height, width, length) and did a quick sketch, that also included noting larger furniture or any particularities within the bedroom. The location of the source was also noted, including measuring its distance from the location of the head (while sleeping). Pictures of all bedrooms were also taken from four different angles.

(3) Three measures were taken in the absence of any noise at the head-side of the bed, about 20 cm above the pillow, in three directions (front, left, right). Each measure took about 10 seconds, and the interval of SPL was noted. Afterwards, the mean value of this interval was taken individually as a proxy for the background level and pooled across all three measurements.

(4) The same measurement procedure was repeated with the switched-on sound source. If participants were ambiguous about their sleep constellation, we determined the 'worst case' (i.e., a constellation with the lowest SPL) and accordingly calibrated the sound source to still produce a SPL of 80 dB to 90 dB in even that situation. In any case, if the measured exposure SPL were below 80dB or unambiguously well above 90dB, the voltage level of the frequency generator was adjusted to alter the SPL. It was made sure that the sound sources did not introduce any audible sounds (e.g., rattling doors or other vibrating objects in the room). For mean SPLs of background- and exposure levels, please refer to the next section.

(5) The sound sources were programmed using a time switch to emit sound for 8 hours, covering the participants' reported habitual bedtime (sleep) interval, including programming potential variations.

(6) Prompters to remind participants of the room constellation were placed in the bedroom. In addition, they received a sleep protocol for checking the constellation every evening before going to bed and for documentation purposes of their bedtime, sleep time, wake-up-time, and to document any deviations. This was mainly done to assure compliance.

1c) On-site constellation details and exposure levels

The average background level measured on-site were within the infrasound verum group (n = 23) 60 dB SPL (measured at the site of the head; in three directions into the room – pooled; SD = 6.2 dB). Exposure levels for the room constellation (i.e., doors and/ or windows open or shut) that was agreed upon with the participant for the time of the study was 88 dB SPL on average (SD = 3.1 dB). The average distance between the head site (bed) and the sources was 2.26 m (SD = 0.54 m). The background level in the infrasound placebo (n = 15) group was 58 dB on average (SD = 4.3 dB), and this did not differ significantly from the infrasound verum group [T (34) = 0.94; *p* = .355]. The average distance between the infrasound placebo sources and the head site (bed) was 2.37 m on average (SD = 0.91 m). Again, this distance did not differ significantly from the source-to-head distance in the verum group [$t(36) = -0.47$; $p = .639$]. The estimated average room volume (based on multiplication of measures of room length, width, and height) in the infrasound verum group was 44.56 m^3 (SD = 13.80 m^3).

Appendix II: Cognitive Test Parameters

Concerning *alertness*, tonic and phasic alertness can be differentiated. Tonic arousal is a good proxy for wakefulness, where the participant is in a state of willfully maintaining his or her attention and accordingly responds quickly to an upcoming event. In the TAP this is implemented as a reaction time (RT) task, where an 'X' is centrally presented on the screen, which the participant is asked to respond to with a button press as fast as possible. RTs are measured under two conditions - with and without a preceding warning tone – in four blocks (total duration: 4.5 minutes). In the present study, the median RTs for trials with vs. without warning signal were used, as recommended, as indicators for tonic and phasic alertness. In addition, the program provides an index of phasic alertness, which contrasts test performance in trials with vs. without warn tone, hence providing an estimate of 'benefit' from the acoustic prompter. In addition, total anticipations are recommended as a measure for disinhibition.

Sustained attention refers to the capacity to concentrate on a routine task for a longer period of time (15 minutes in the case of the test). In the TAP, a successive discrimination task is implemented. Participants are instructed to press a button when two stimuli with the same form (e.g., an oval) appear after one another. Out of 450 stimuli, only 54 are targets, and these are evenly distributed across the task, within three 5-minute-intervals. Hence, participants need to sustain their concentration in order not to miss targets. We used the number of omissions as indicator for potential changes in sustained attention.

Flexibility is the ability to redirect attention from one to another object, modality, or task. It is a heterogeneous construct, ranging from willfully shifting the sensory focus to strategic control of complex coordinated behavior. In the TAP, a letter and a number are presented left and right (or reversely) simultaneously on the screen. The participant has the instruction to shift to which stimulus type he or she must respond for each trial, starting responding to the side (left or right button) where the letter is presented, then shifting to responding to the side where the number is presented, and so on, for 100 trials with a duration

of about 3 minutes. Most important parameters are the total performance index and the speed-accuracy (trade-off) index. The total performance index integrates both the correctness and speed of reactions, where positive values indicate above-average fast and correct reactions. Negative values accordingly indicate below-average performance (high error rate, slow reactions). If the performance is (approximately) zero, this indicates average total performance. A negative speed-accuracy index denotes a relatively high error rate and fast responses (speed strategy), whereas positive values indicate low error rates but long RTs (accuracy strategy).

Divided attention refers to the ability to simultaneously attend to concurrent tasks. In the TAP this is operationalized in a task where participants are simultaneously presented with stimuli of two different modalities: vision and hearing. While the participant must discriminate between, and respond to, visual target stimuli, at the same time successive high and low beep tones are presented. Responses are required to either when a visual target stimulus (20 out of 175 stimuli) or two successive high or low tones (20 pairs out of 287 tones) are presented. The total task takes about 3.5 minutes. The number of omitted targets is the most important test parameter, as indicating the inability to attend to both modalities simultaneously.

Incompatibility effects occur in conflict situations, where divergent information generates a conflict in reaction tendencies. The TAP applies the Simon-paradigm. The aim of the task is to respond with the correct hand, as indicated by the direction that is pointed out by an arrow. The arrow however not only points into the direction of the hand with which participants have to react, it also appears on the right vs. left side of the screen, creating compatible (e.g. pointing left, appearing on the left side) vs. incompatible (e.g. pointing left, appearing on the right side) information. Incompatible information results in conflicting reaction tendencies, whereby the irrelevant, incompatible information needs to be discarded. Incompatibility is manifest in slower RTs, while compatibility leads to faster RTs. In total, there are 60 trials, of which 15 are compatible and 15 incompatible on each side (left, right). Of interest are the parameters: errors in incompatible conditions and validity x side interactions.

The latter quantifies the incompatibility effect, with lower values denoting a stronger and higher values a weaker effect.

Covert shift of attention describes the ability to shift the attentional focus without making eye movements (i.e., endogenous attentional shift). A target stimulus appears at the left or right side of the screen, whereby its appearance is preceded by a cue arrow-stimulus, presented centrally on the screen, which points to the congruent (where the stimulus is truly going to appear) or incongruent side (opposed to where the stimulus is going to appear). The task includes 100 trials, of which 20 are invalid and 80 are valid cued trials. Particularly meaningful for interpreting the task performance is the validity x side index, which indicates the shift of attention ability. Lower values indicate more pronounced problems to willfully re-orient attention after an invalid cue while higher values indicate better performance.

The *GoNogo* task measures the inhibition capacity of the respondent. A primary, externally triggered impulsive reaction tendency needs to be suppressed in favor of an internally controlled reaction. In the TAP this is implemented by presenting a successive sequence of 5 different types of stimuli, out of which 2 types are targets. All stimuli look relatively similar, which is why oftentimes responses need to be suppressed. It is recommended to evaluate the number errors as a marker of lower inhibition.

Appendix III – Descriptive pre-post data (means and standard deviations) for all behavioral variables

Variable	Infrasound verum – T1 (n = 23)	Infrasound verum – T2 (n = 23)	Infrasound placebo – T1 (n=15)	Infrasound placebo – T2 (n=15)
Sensitivity				
Low frequency sensitivity	6.39 (7.97)	8.43 (7.33)	6.80 (7.12)	6.80 (6.98)
Normal sound sensitivity	46.6 (14.6)	46.9 (17.5)	49.4 (17.9)	50.1 (15.6)
Symptoms and Sleep				
BSI total	10.5 (8.70)	11.2 (10.1)	17.5 (14.7)	18.8 (16.6)
BSI somatization	1.00 (1.38)	1.22 (1.73)	1.40 (1.30)	0.87 (1.13)
BSI depressive symptoms	0.96 (1.22)	1.13 (1.58)	1.07 (1.71)	1.67 (2.09)
BSI anxiety symptoms	1.52 (1.78)	1.43 (1.80)	2.47 (2.00)	1.80 (1.61)
ESS sleepiness	8.70 (4.08)	8.22 (4.35)	9.87 (3.78)	7.07 (4.45)
PSQI – sleep quality (total)	5.53 (1.39)	5.78 (1.56)	6.57 (2.82)	5.92 (1.62)
PSS perceived stress	21.6 (5.25)	20.7 (5.34)	23.5 (8.04)	25.2 (6.38)
Alertness				
Median RTs tonic arousal	232.9 (25.7)	227.7 (21.2)	244.8 (41.4)	248.1 (41.4)
Median RTs phasic arousal	234.3 (25.0)	223.0 (26.8)	245.3 (49.2)	235.3 (37.4)
Phasic alertness index	-.006 (.060)	.024 (.072)	.001 (.078)	.052 (.072)
Anticipations after warn tone	0.70 (1.06)	1.39 (1.70)	1.27 (2.52)	0.73 (1.28)
Sustained Attention (WM)				
Omissions	4.83 (5.05)	4.30 (5.30)	3.60 (3.83)	4.27 (5.56)
Flexibility				
Speed-accuracy index	-3.49 (7.70)	-3.33 (9.05)	6.03 (8.33)	2.08 (7.77)
Total performance index	8.19 (8.07)	9.82 (6.37)	-1.13 (7.56)	1.99 (8.38)
Divided Attention				
Omissions (total)	1.43 (1.83)	1.35 (1.56)	2.20 (3.73)	2.27 (2.94)
Incompatibility				
Incompatibility effect	3.48 (2.93)	5.18 (5.67)	8.10 (9.14)	6.46 (8.49)
Errors incompatible	2.91 (5.47)	2.09 (1.73)	2.00 (2.04)	3.47 (7.59)
Covert Shift of Attention				
Validity x side index	2.06 (2.57)	1.36 (1.79)	1.24 (1.88)	3.29 (5.39)
GoNoGo				
Errors (total)	0.39 (0.58)	0.22 (0.42)	1.07 (3.33)	1.00 (2.80)
Personality				
Openness	15.9 (4.55)	15.4 (4.01)	16.1 (3.65)	16.1 (4.03)
Neuroticism	5.74 (3.61)	5.00 (3.70)	7.47 (4.39)	8.47 (5.07)
Conscientiousness	19.2 (2.99)	19.8 (2.87)	18.5 (2.47)	18.7 (2.69)
Extraversion	15.2 (2.50)	14.9 (2.59)	14.7 (4.13)	13.5 (4.49)
Agreeableness	4.57 (3.64)	4.90 (4.30)	4.73 (3.58)	5.80 (4.20)

Appendix IV – Results and brief discussion concerning regional white matter volume change from pre-to-post in verum relative to placebo

For details on the analysis procedure, please refer to the main paper. There was one cluster of significant increase in regional white matter volume (rWMV) in infrasound verum relative to placebo pertaining to the right middle frontal gyrus (rMFG; 35, 24, 41; t = 5.29, k = 214). Two clusters of significant decline in rWMV were also identified; pertaining to the right frontal operculum (rFOp; 44, 14, 2; t = 4.40, k = 160) and right middle cingulate gyrus (rMCgG; 0, 9, 42; t = 4.07, k = 198). See Appendix Figure 3 for a graphical depiction of the clusters.

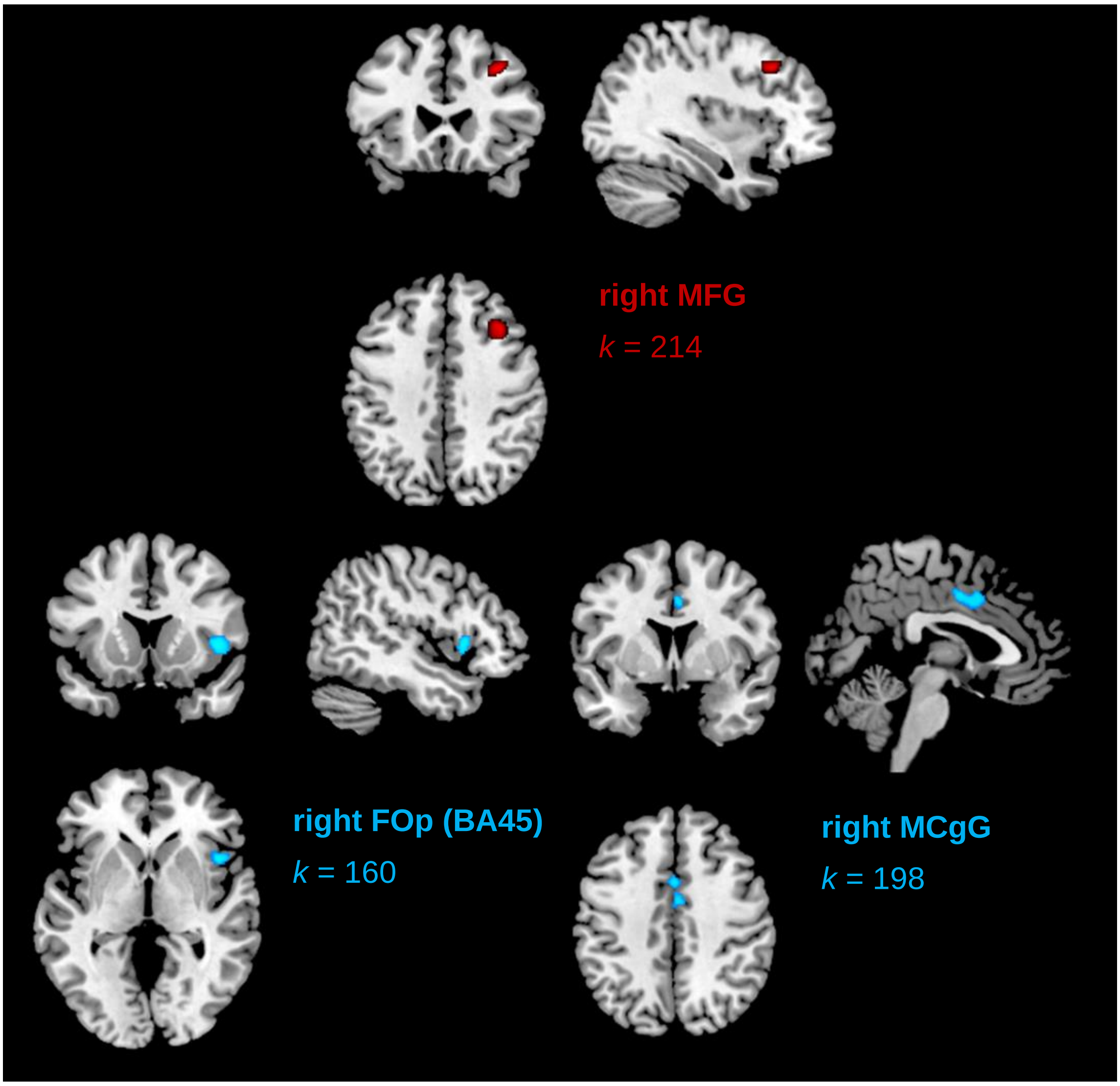

Appendix Figure 3. Graphical depiction of identified significant clusters in the VBM analysis searching for increases or decreases in rWMV in the infrasound verum condition (relative to the sham condition) from pre-to-post; increases are depicted in red, decreases in blue.

The cluster of rWMV decline in the posterior operculum (roughly pertaining to BA45) is also part of a speech-relevant area (both right and left-handed individuals were recruited – hence lateralization might not play such a strong role) which might further strengthen the finding concerning regional grey matter volume decline (rGMV) in left angular gyrus (see main paper) and hence potential relevance to investigate speech-related functions as potentially affected by infrasound. The decline in the middle cingulate gyrus, which appears to be located rather at the posterior portion of the mid cingulum as part of the premotor area, which involves neurons functionally attuned to respond to head and body movements and detection of direction and force [1]. Finally, the middle frontal gyrus (MFG) as part of a larger network (including premotor cortex, posterior parietal cortex, medial frontal cortex and right inferior frontal cortex) has been identified as relevant to modulating attentional shift between endogenous (top-down control) and exogenous attention (bottom-up control),[2] and has been assigned an important role in reverting from exogenous to endogenous control in a lesion study with a patient with right MFG resection [3]. Future studies will need to reveal, what the identified changes imply on a functional and behavioral level.

Appendix References

1. Vogt BA. Midcingulate cortex: Structure, connections, homologies, functions and diseases. *J. Chem. Neuroanat*. **74**, 28–46 (2016).

2. Peelen MV, Heslenfeld DJ, Theeuwes J. Endogenous and Exogenous Attention Shifts are Mediated by the Same Large-Scale Neural Network. *NeuroImage* **22**(2), 822–30 (2004).

3. Japee S, Holiday K, Satyshur MD, Mukai I, Ungerleider LG. A role of right middle frontal gyrus in reorienting of attention: a case study. *Front. Syst. Neurosci.* **9**(23) (2015).
